# Magnetic Particles for Advanced Molecular Diagnosis

**DOI:** 10.3390/ma12132158

**Published:** 2019-07-05

**Authors:** Cristina Chircov, Alexandru Mihai Grumezescu, Alina Maria Holban

**Affiliations:** 1 Faculty of Applied Chemistry and Materials Science, Politehnica University of Bucharest, 011061 Bucharest, Romania; 2 Microbiology Immunology Department, Faculty of Biology, University of Bucharest, 050095 Bucharest, Romania

**Keywords:** molecular diagnostics, gene expression, biological markers, molecular genetics, cancer, infectious disease, magnetic particles

## Abstract

Molecular diagnosis is the field that aims to develop nucleic-acid-based analytical methods for biological markers and gene expression assessments by combining laboratory medicine and molecular genetics. As it gradually becomes a clinical reality, molecular diagnosis could benefit from improvements resulting from thorough studies that could enhance the accuracy of these methods. The application of magnetic particles in molecular diagnosis tools has led to tremendous breakthroughs in terms of specificity, sensitivity, and discrimination in bioassays. Therefore, the aim of this review is to highlight the principles involved in the implementation of magnetic particles for sample preparation and targeted analyte isolation, purification, and extraction. Furthermore, the most recent advancements in the area of cancer and infectious disease diagnosis are presented, with an emphasis on screening and early stage detection.

## 1. Introduction

The practice of medicine is currently focusing on the development of quick and accurate tests for assessing the health of an individual at the cellular and molecular levels. Such tests have been previously reported as point of care devices, which are instruments designed to perform analytical and diagnostic tests at the site of interest, including the hospital, but also in the field [1,2,3,4]. All body processes, both physiological and pathological, are driven by specific genetic sequences and proteins. Therefore, the role of genetic mutations and risk factors that are causing or contributing to the disease, often termed biomarkers, must be known [5,6]. Besides those diseases with clear heritable components, such as cystic fibrosis, phenylketonuria, hemophilia, or even cancer, genetic material is involved in all disease processes, through the susceptibility of an individual to a specific disease as a consequence of the various differences in our genetic code [7,8].

Accordingly, molecular diagnostics has emerged as the field that combines laboratory medicine and molecular genetics [8,9] to develop nucleic-acid-based analytical methods for biological marker and gene expression assessments [8,10]. Specifically, molecular diagnostics encompasses a series of techniques which aim to identify variations at the gene, RNA, or protein levels [6]. Therefore, nucleic-acid-based testing has become a unique diagnostic tool for prognosis, detection, diagnosis, sub-classification, and monitoring diseases [6,9], but it could also be applied for developing personalized therapies based on an individual’s genomic makeup [8,10,11,12]. Additionally, molecular diagnostics offers a path for carrier analysis and prenatal diagnosis, which could provide reliable references for genetic counseling [8,13]. Another application of molecular diagnostics involves the detection and quantification of specific bacteria, viruses, and infectious diseases at earlier stages than conventional diagnostics can provide [6,14,15].

**Figure 1:**
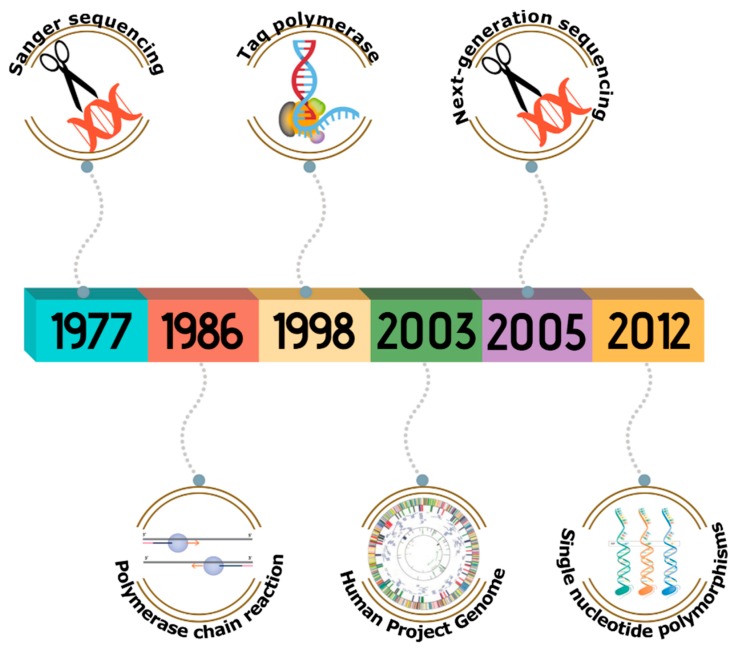
A brief history of molecular diagnostics.

The second half of the twentieth century represented the birth of molecular diagnostics, through the discovery of the Sanger sequencing method in 1977, also known as the chain termination method, which provides high-quality sequencing for long fragments of DNA of up to 900 bp [16,17]; and the polymerase chain reaction in 1986 by Mullis et al., which allowed for the production of many copies of a DNA target region [9,18,19]. Other breakthroughs in the field of molecular biology include the discovery of the thermostable Taq polymerase in 1998 [19], the completion of the Human Genome Project in 2003 [20], the first next-generation sequencing platform launched by the 454 Life Science company in 2005 [17], and the use of hybridization tests for the recognition of specific single nucleotide polymorphisms causing an individual’s disease in 2012 (Figure 1) [21].

Although it has firstly involved targeted searches in one specific gene or region, the field of molecular diagnostics is growing rapidly towards techniques that allow for the simultaneous survey of the entire genome [22]. Moreover, early methodologies focused on the indirect detection of mutations in common disorders, such as hemoglobinopathies and cystic fibrosis, which were laborious, required large amounts of nucleic acid, and often led to uninterpretable results [23]. However, several criteria, such as the type of nucleic acid and specimen, number of mutations, and reliability of the method, must be considered when choosing a suitable method [8]. The first molecular diagnostics tests approved by the Food and Drug Administration were probe techniques, which are still currently used, as they fill important niches [24].

Nucleic acid testing generally involves techniques for DNA and/or RNA isolation, amplification, detection, and discrimination [25]. Nucleic acid isolation usually involves lysis (i.e., chemical, enzymatic, mechanical, or physical techniques), separation, purification, and concentration techniques [26]. Amplification refers to the techniques employed for increasing the amount of the target nucleic acid, the detection signal, or the probes, namely polymerase chain reaction, transcription-mediated amplification, whole genome amplification, antisense RNA amplification, etc. [25]. Variant detection approaches can be categorized into genetic screening methods, for the detection of specific genomic variants (e.g., amplification refractory mutation system, allele-specific oligonucleotide probes, oligonucleotide ligation assays, competitive oligopriming, and chemical and enzymatic cleavage), and genetic scanning methods, which aim to detect every genomic variant within the amplified fragment (e.g., single-strand conformation polymorphism, heteroduplex analysis, and denaturing- and temperature-gradient gel electrophoresis) [19]. Other classification includes enzymatic-based, electrophoretic-based, and solid phase-based techniques [9]. Discrimination techniques can be divided into electrophoretic methods for physical nucleic acid separation based on molecular size or shape, alternative approaches that determine size, base content, or sequences without electrophoresis, such as high-performance liquid chromatography, mass spectrometry, and pyrosequencing, and hybridization assays for specific nucleic acid identification by annealing or melting complementary nucleic acids [25].

These techniques have set the standards for the development of high-throughput systems, such as microarrays for large-scale single nucleotide polymorphism genotyping and DNA diagnostics of inherited, acquired, or infectious diseases [19]. However, as molecular diagnostics is gradually becoming a clinical reality [9], thorough studies for improving the accuracy of these methods are still crucial. The aim of this paper is to provide an overview of the implications of magnetic particles in advancing molecular diagnosis tools.

## 2. Magnetic Particles in Diagnosis 

Fast, selective, and accurate disease diagnosis based on molecular tools is fundamental for providing more precise preliminary medical assessments. However, as biological samples are highly complex, due to the presence of multiple components, the specific target entity must be isolated from the raw sample prior to analysis [27]. Traditional methods for the separation of target biomolecules, based on fluid-phase methods, such as centrifugation and filtration, are laborious and time-consuming, and they usually result in the degradation of the biological material [28,29]. An alternative to overcome these limitations might be the utilization of sorptive extraction techniques, i.e., magnetic separation, which involves the magnetophoresis phenomenon, through which the magnetic entities will migrate relative to their non-magnetic surrounding environment under a heterogenous magnetic field. Specifically, by applying an external magnetic field that will generate a magnetic field gradient, the target entities are magnetically isolated from the sample, either through intrinsic magnetic characteristics or by labelling with magnetic materials [27].

**Figure 2:**
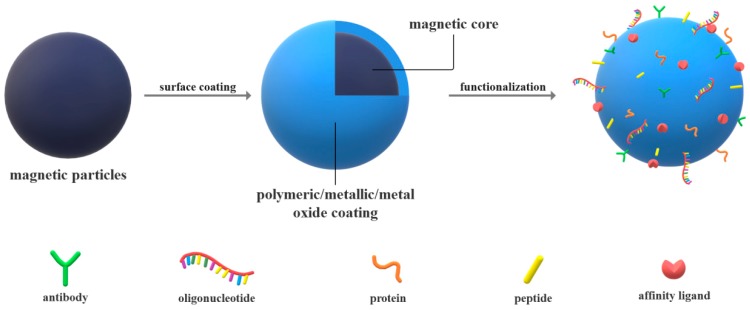
The process of synthetizing magnetic particles for molecular diagnosis.

Owing to their advantageous surface properties and versatility, magnetic particles have been studied for a variety of biomedical applications, from targeted drug delivery, bioimaging systems, and hyperthermia for cancer therapy, to separation techniques and bioassays, such as blood screening, cell labeling, sorting, and identification, nucleic acid, bacteria, and virus capture, isolation, concentration, and detection, and immunosensors and immunoassays [30,31,32,33,34,35]. Magnetic particles usually consist of oxides of iron, nickel, and cobalt, or other elements combining several metals, such as zinc, copper, strontium, and barium [30,36,37,38,39]. There are various top-down and bottom-up synthesis methods to prepare spherical particles with a narrow size distribution, including ball milling, co-precipitation, hydrothermal synthesis, thermal decomposition, laser ablation, microemulsion, chemical vapor deposition, arc discharge methods, flame spray synthesis, and biosynthesis methods [36,40,41,42]. Although they are characterized by larger saturation magnetization, iron, cobalt, and nickel particles are toxic; therefore, for biomedical applications, iron oxide particles in the form of magnetite or maghemite are used [40,43]. Moreover, considering their tendency to agglomeration due to van der Waal and dipole–dipole attractions, and their sensitivity to oxygen, pH, and salts in the environment, the surface of these particles must be modified to increase their physical and chemical stability by providing steric and Coulomb repulsions [40,41]. Nanoparticle surface coatings are usually performed in the synthesis stage, and they involve polymeric coatings using different natural and synthetic structures, such as chitosan, dextran, cellulose, polyethylene glycol, polyvinyl alcohol, polystyrene, or polyethylene imine, liposomes and micelles utilization, core-shell structures using silica, and metallic coatings, such as gadolinium, or other hybrid materials [36,43,44,45,46]. Additionally, magnetic particles can be functionalized with different compounds in order to provide functional groups for further bioactive molecule conjugations [41] through different techniques, such as direct binding, Hong’s method, or bioremediation [40]. Specifically, the functionalization of magnetic particles allows for the attachment of ligands and receptors on their surface and the binding of biomolecules, such as monoclonal antibodies, nucleic acids, streptavidin, proteins, and peptides, to ensure specific interactions with the target molecules (Figure 2) [30]. In this context, the manufacture of magnetic particles does not generate any byproducts that are hazardous to the environment or to human health. Additionally, they can be further reused for multiple analyses, which is highly advantageous due to cost and time reductions. Therefore, magnetic particles represent a safe and versatile platform for bioassays that are otherwise highly laborious.

In the context of molecular diagnosis, magnetic particles have been applied for mixing fluids, selectively capturing, concentrating, transferring, and labelling targeted analytes, performing stringency and washing steps, and probing biophysical properties of analytes [37,38,39,46,47,48]. In this tasks, magnetic separation offers a variety of advantages, including high-throughput, low costs, energy consumption efficiency, increased specificity, stability, and sensitivity [27,31,49], and it has been intensively used, since most biological materials are not susceptible to magnetic fields and the magnetic particles can be easily separated from the reaction mixture and re-dispersed after the removal of the magnetic field [30]. Further, the two main applications of magnetic particles in molecular diagnosis will be discussed, specifically for sample preparation and extraction and for molecular detection and readout systems [46].

Sample preparation is the initial and a key step for any analytical workflow that aims to qualitatively and quantitatively determine the presence of trace analytes in complicated matrices [50]. Specifically, sample preparation represents the process of reducing the complexity of a biological sample by removing interferents [51]. The most common biological samples are liquid, such as urine, plasma, serum, and saliva, or solid, including tissue sections, such as hair or nail [52]. Although centrifugation and protein precipitation are the most popular methods for the separation of biological samples, advanced techniques have been further developed, from pressurized fluids to microwave-assisted systems and microextraction techniques, since sample preparation also aims to perform additional tasks, such as analyte concentration, enrichment, and derivatization [53]. However, the existing kits and instruments for sample preparation from companies such as Invitrogen, Roche, Qiagen, and bioMerieux range in cost from $15,000 to $80,000 [54]. As high resolution and specificity, easy operation, and short analysis time are desirable features for the separation of biological samples [28,55], sorptive extraction procedures have been employed as manually operated methods. 

With the rapid development of nanotechnology in materials science, various nanomaterials useful for analytical chemistry have been manufactured. Their potential for rapid and efficient sample preparation is based on their high specific area, increased surface activity, and unique physicochemical properties [50]. Furthermore, magnetic nanomaterials play a fundamental role in this area, as they allow for the magnetic separation of biological targets from raw samples and reduce the non-specific adsorption of interfering biomolecules without damaging the sample [28,29]. This is of key importance for the development of point of care tests or devices, as they offer a more facile platform for extracting the biocompounds of interest from the patient’s sample. In this manner, they could revolutionize the field by eliminating the need to use equipment that is only available in specialized laboratories. Additionally, this technique does not require specially trained personnel, thus reducing the costs even more.

**Figure 3:**
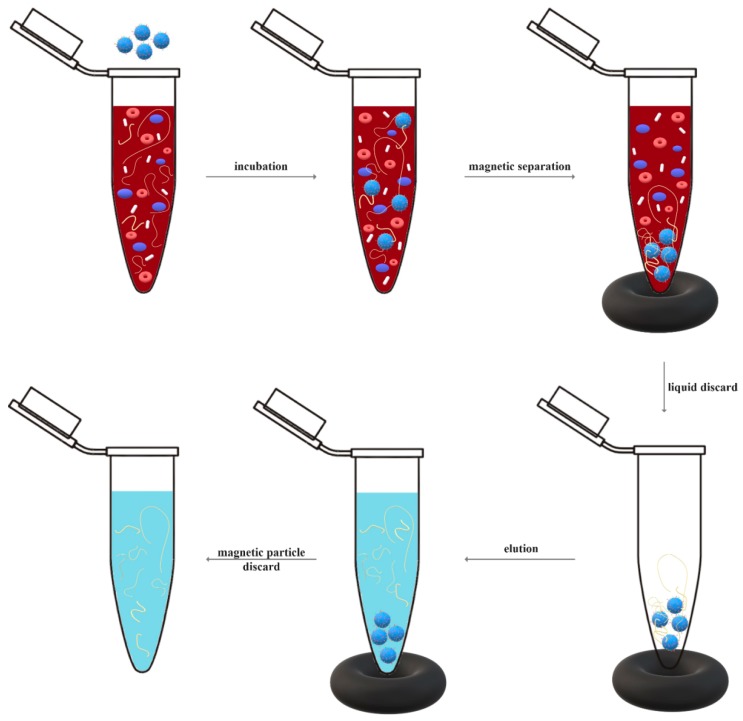
A schematic representation of the steps involved in magnetic separation.

The general steps involved in the extraction of target analytes are effective cell or tissue disruption, the removal of membrane proteins, lipids, and other contamination through the denaturation of nucleoprotein complexes and the inactivation of nucleases, and nucleic acid purification and concentration [55,56]. In the case of magnetic separation, the previously modified ferromagnetic or superparamagnetic particles are added to the sample and incubated for a certain amount of time, to allow for the interaction with the target analytes through affinity adsorptions or antibody–antigen or hydrophobic interactions. As nucleic acids are polyanionic molecules with numerous phosphate groups, the electrostatic interactions could be increased through functionalization with positively charged species, such as aminosilanes; similarly, the immobilization of specific oligonucleotide sequences could allow for the affinity capture of nucleic acids through the hybridization of complementary sequences. Another strategy involves the interaction between the target analytes and the affinity ligands and, after the removal of the excess ligands, the capture of the resulting complexes by the magnetic particles through intermediary ligands. Subsequently, the particles are separated from the sample by applying a magnet to the outside of the vessel wall. The resulting analytes are eluted from the particles and subjected to further analyses (Figure 3) [29]. Further, in order to quantify genetically modified organism content and pathogens within the samples, nucleic acid concentrations can be measured by evaluating the intensity of a band on an agarose gel, determining the ultraviolet absorbance of fluorescent nucleic acid-binding dyes, or through the real time PCR technique [57].

The other direction of applications in molecular diagnosis is represented by the molecular detection of biomolecules labeled with magnetic particles. Commonly used biosensors based on magnetic particles with various sensing properties include magnetic-particle-relaxation-based sensors, magnetoresistive sensors, and magnetic relaxation sensors [44]. Moreover, there are many technologies available for magnetic particle detection, such as superconducting quantum interference devices, atomic magnetometers, and diamond-based magnetometers. Of these, atomic magnetometers are the most sensitive to magnetic fields, and allow for various non-invasive sensing modalities operated in ambient conditions [58]. The advances in the field of biosensors could revolutionize the fast and sensitive quantification of biomarkers for diagnosing Alzheimer’s disease, chronic kidney disease, diabetes, liver diseases, tuberculosis, atherosclerosis, sepsis, and cancer [59]. Additionally, magnetic particles can be implemented into electrodes for the specific detection of chemical and biochemical compounds. The magnetic-particle-modified electrodes are highly advantageous, owing to their high selectivity and activity and strong adsorption capacity [60]. Nevertheless, magnetic separation poses a series of limitations which must be overcome in order to improve its efficiency. Specifically, magnetic cell sorting is characterized by a medium throughput of 10^9^ cells/hour, and, to reduce the detection time and enhance the sensitivity, larger sample volumes are required. Moreover, as the resulting culture is not highly pure, magnetic separation must often be coupled with other methods to improve detection [61,62]. Another limitation of the method is represented by the tendency of magnetic particles to aggregate, thus reducing the capacity to bind to the targeted analytes.

Recent years have witnessed a tremendous interest in applying microfluidics for sample preparation, cell separation, cancer diagnosis, and delivery and screening of therapeutic drugs. Microfluidic systems, known as lab-on-a-chip, biochips, or bio-microelectromechanical systems integrate and implement multilaboratory functions, such as chemical synthesis, biochemical operations, or nucleic acid sequencing, onto a single microdevice [46,63,64]. Such systems offer the advantages of limited sample consumption by working with extremely small volumes, low costs, rapid sample screening, portability, and higher sensitivity [63,64,65,66,67,68]. Microfluidic active manipulation techniques generally exploit external forces, including optical, acoustic, dielectrophoretic, and magnetic forces. Each system is characterized by different advantages and disadvantages, i.e., optical force can capture single particles and cells, but can generate Joule heat, acoustic force is limited by its low resolution, dielectrophoresis achieves high cell throughput, but the dissolved ions and surface potential could lead to cell damage; by contrast, magnetic manipulation is easy to control through the external magnetic field, does not produce additional heat, and does not require expensive external systems [65]. For these reasons, magnetic particles have been widely integrated into biological tests for capturing, transporting, labeling, and detecting biomolecules within samples [31]. The process of bioseparation is similar to the magnetic separation previously described. Hence, magnetic particles serve as supports for nucleic acid hybridization, intercalation, and purification, allowing for simple and rapid analyses [46]. Furthermore, a recently developed magnetic-particle-based microfluidic device for point of care assays is the centrifugal microfluidic device, which is a compact disc that incorporates interconnecting channels and chambers. Such devices, also termed lab-on-a-disk systems, have the advantage of using a motor that will generate centrifugal forces, acting as liquid pumps that will further produce a force gradient, Coriolis forces, which control the direction-specific liquid pumping, and Euler forces, which create turbulences during mixing. In this manner, components could be separated by density, air bubbles are removed, and liquids are pumped without contact with external hardware [68]. Hence, the combination of magnetic particles and microfluidics represents a fundamental step toward the clinical reality of point of care devices, as these systems could be easily used outside laboratory facilities. Moreover, such systems could be developed in such a way that patients could directly use them at home to continuously monitor their disease without the need for transportation to a health care facility.

There are several parameters that must be considered when designing magnetic-particle-based molecular diagnosis applications. Specifically, these parameters affect the outcome of the analysis, and they include the size and composition of both the magnetic core and the shell, the surface chemistry, and the magnetic responses to the sensor [69]. While microfluidics could offer a potential solution for the previously mentioned limitations of magnetic separation, there is still room for improvement regarding the physicochemical properties of magnetic particles.

## 3. Cancer Diagnosis

Defined by the World Health Organization (WHO) as the ‘presumptive identification of unrecognized disease or defect by the application of tests, examinations or other procedures, which can be applied rapidly to the target population’ [70], cancer screening is an important factor that contributes to the reduction of the incidence, morbidity, and mortality of cancer. In the case of cervical cancer, the effects of screening are considerably dramatic, with a decrease in mortality of over 80% in U.S. after the introduction of Pap smears [71,72]. Similarly, early detection is a critical factor in patient survival rate, as for detection of the primary tumor through routine cancer screenings, the prognosis is usually fatal [73]. Thus, the common goal of cancer screening and early detection is to detect the malignancy or preneoplastic states prior to the symptom onset, as the treatment is the most effective at this point [74].

Biomarkers are biological molecular entities that can be objectively measured and are associated with physiological functions, disease progression, treatment efficiency, and adverse effects to a therapeutic agent [75,76]. Biomarkers can be classified based on a range of parameters, namely their characteristics, including imaging biomarkers and molecular biomarkers (nucleic-acid-based biomarkers, such as gene mutations or polymorphisms and quantitative gene expression analysis, peptides, proteins, lipids metabolites, and other small molecules), and their applications, including diagnostic biomarkers, disease prognosis biomarkers, disease staging biomarkers, and clinical response monitoring biomarkers [77]. Cancer biomarkers are biological molecules produced by cancer cells or body tissues as a response to cancer development, and they indicate tumor progression [75,76]. Nucleic acids, proteins, peptides, oncofetal antigens, cytokeratins, carbohydrates, or hormones can serve as biomarkers, and they can be found in biological fluids, including blood, saliva, urine, stool, sputum, and cerebrospinal fluid, as circulating cancer cells or cell-free cancer DNA, which enters by direct release from the original tissue through the necrotic tumor cells by phagocytosis or cell lysis [73,75,76,78]. Thus, by measuring the amount of biomarkers, it is possible to predict the risk of cancer development, screen for early cancer detection, diagnose patients experiencing cancer symptoms, provide a prognosis, and predict and monitor cancer therapy responses, pharmacodynamics, and tumor recurrence [76,78,79]. Although there is a tremendous scientific focus on the discovery of novel cancer biomarkers [80,81,82,83], to date, most biomarkers have proven poor accuracy and efficacy [78].

Magnetic particles have proved their important potential in cancer imaging, diagnosis, and treatment. Conventional cancer imaging techniques include magnetic resonance imaging, magneto-acoustic tomography, computed tomography, and near-infrared imaging. Although they offer the possibility of non-invasive diagnosis, their application is limited in the context of detecting subtle invasion, micrometastases, and early stage detection [84,85]. Consequently, magnetic particle imaging is a tracer imaging modality that directly measures superparamagnetic iron oxide nanoparticles based on their vivo relaxation dynamics. This technique is characterized by several advantages, including high image contrast, signal linear with the tracer concentration, no depth attenuations, high sensitivity, high temporal resolution, and safety, as it does not involve ionizing radiation [86,87]. Owing to their characteristics, such as small size and high specific area, magnetic particles offer a promising alternative for cancer diagnosis. Hence, by accurately binding to specific molecules from the biological sample, magnetic particles could aid the detection of trace analytes that are relevant to cancer diagnosis. Thereupon, they could allow for the early detection of malignancy prior to the onset of symptoms, and offer the possibility of developing novel cancer therapies with higher survival rates.

In the context of molecular diagnosis, there are many studies described in the literature reporting the use of magnetic particles for the specific detection of cancer biomarkers. Thus, the development of a next generation, aptamer-based bio-barcode assay to detect cytochrome-c, a biomarker released from cancer cells, has been reported. Specifically, magnetic microparticles coated with capturing antibodies and a specific aptamer against cytochrome-c led to the formation of sandwich structures. In this manner, the detection process can be completed within three hours, thus providing a fast, sensitive, and robust tool for anti-cancer drug screening [88]. Similarly, a two-step assay based on the isolation of metallothioneins, proteins known to serve significant roles in carcinogenesis, using functionalized paramagnetic particles and their subsequent electrochemical analysis, has resulted in a simple, inexpensive, error-free, and fully automated technique that could be implemented in an instrument for monitoring carcinogenesis and metallothionein-related chemoresistance [89]. Human epididymis protein 4 is a new biomarker approved by the Food and Drug Administration, which has received considerable attention owing to its capacity for the diagnosis of epithelial ovarian cancer [90]. By coating magnetic particles with alkaline phosphatase antibody, a sensitive and specific method for the determination of human epididymis protein 4 by chemiluminescence immunoassay has been developed [91]. Furthermore, a novel method for the separation and detection of circulating ovarian cancer cells using whole blood samples has been demonstrated. Specifically, iron oxide nanoparticles synthesized through a pyrolysis-based method and further functionalized with folic acid were used for the separation of ovarian cells from the female, fresh, whole blood samples. Results showed that the isolated cells over-expressed the human epididymis protein 4 biomarker, which is specific for ovarian cancer [92]. Similarly, functionalized magnetic particles have been used for the separation of CD133+ expressing cells from the peripheral blood of patients with gastric adenocarcinoma [93]. As the alpha-fetoprotein biomarker offers limited sensitivity and specificity for the early detection of hepatocellular carcinoma, the combination with des-gamma-carboxy prothrombin is necessary. In this context, one study reported the synthesis of iron oxide magnetic nanoparticles coated with anti-des-gamma-carboxy prothrombin antibodies for immunomagnetic reduction assay, with results proving its potential for future clinical applications [94]. Additionally, a microfluidic-based strategy to develop hierarchical silica–magnetic microflowers with multilayered structures from a miniaturized five-run spiral-shaped microreactor, and to subsequently conjugate the EpCAM antibody on the surface, has been shown to exhibit high capture rates toward MCF-7 tumor cells from whole blood samples [95].

In addition, magnetic particles have proven to be efficient drug carriers for cancer therapy, as they tend to accumulate at specific locations through the application of an external magnetic field [85]. Moreover, they have been used for the fast, effective, and site-specific delivery of biotherapeutics, such as therapeutic cells, proteins, and nucleic acids [96]. Hypocrellin B-loaded magnetic mesoporous silica nanoparticles cloaked with red blood cell membranes have been shown to avoid immune clearance and accumulate at the tumor site through the action of the magnetic field, leading to necrosis of the tumor tissue [97]. Other examples of magnetic-particle-based systems for the delivery of anti-cancer drugs and additional hyperthermia therapy include magnetic particles containing artemisinin, a compound with anti-proliferative and anti-angiogenic properties in cancer cells [98], curcumin-loaded dendrimer-modified citric acid-coated iron oxide nanoparticles [99], cetuximab-loaded core shell iron oxide and gold nanoparticles [100], and folic-acid-functionalized cobalt-ferrite magnetic nanoparticles containing doxorubicin [101]. Moreover, magnetic particles could also be applied in gene therapy, as Bag-1, a positive regulator of the anti-apoptotic Bcl-2 gene, which is generally over-expressed in colon cancer, was immobilized onto magnetic gold nanoparticles along with siRNA for cell transfection, which could be potentially used in cancer treatment through siRNA silencing method [102].

## 4. Diagnosis of Infectious Diseases

Responsible for highly diverse processes such as photosynthesis, nitrogen fixation, vitamin production, and organic matter decomposition, microorganisms play crucial roles in the existence of humans. While there are some highly specialized microorganisms that can survive in harsh environments, root-colonizing bacteria rely on the abundant resources provided by higher organisms [103]. In some cases, the delicate balance between pathogens and host immune system shifts to the benefit of pathogens, leading to immune deficiency states [104]. Therefore, infectious diseases can be defined as disorders caused by pathogenic microorganisms, such as viruses, bacteria, parasites, or fungi, which can be directly or indirectly spread from one individual to another [105]. For this reason, they are often considered as a war against microbes [106].

Infectious diseases and their worldwide spread have become a serious public health concern, as they affect millions of lives daily [107,108]. The effective, early diagnosis of infectious diseases is a major issue, since rapid diagnostic tools could reduce the incidence of these disorders and the risk of developing antimicrobial resistance, avoid overtreatment due to the administration of inadequate treatment, allow for distinction between pathogens that present similar symptoms, and prevent deterioration and further spreading [107,108,109]. Standard clinical diagnosis techniques include invasive detection through biopsy or endoscopy and biomarker-based diagnostic tools, such as microscopy, cultures, enzyme-linked immunosorbent assay, lateral flow assay, and polymerase chain reaction [107,109]. Recently, advancements in nanotechnology, especially in the design of magnetic nanomaterials, have provided a new platform for the diagnosis and treatment of infectious diseases, as they hold great potential for the selective detection of microorganisms [108]. In this manner, by integrating magnetic particles into bioassays and biosensing devices, rapid diagnosis could be achieved without sample pre-enrichment, purification, or pre-treatment steps. Thus, magnetic particles could be applied for the specific isolation of biomarkers related to infectious diseases [107]. Such bioassays could be widely applied in underdeveloped countries, where lethal infections due to environmental pathogens or lack of proper vaccinations are a serious cause for concern. Moreover, as there is a limited number of health care facilities in such countries, magnetic-particle-based point of care devices represent a solution for the accurate diagnosis and appropriate treatment of infectious diseases, thus reducing the associated morbidity and mortality.

There have been several studies in the literature reporting the use of magnetic particles for the detection of viruses. Specifically, hepatitis A virus and hepatitis B virus were captured and detected using carboxyl-derivatized magnetic beads [110] and magnetic digital microfluidic systems [111], respectively, with improved specificity and virus liberation from the matrix. Furthermore, one study reported targeting and isolation of the influenza virus using erythrocyte-membrane-cloaked nanoparticles modified with magnetic functionalities by encapsulating superparamagnetic iron oxide nanoparticles. Besides the improvement of diagnosis, these systems prove the potential of membrane cloaking mechanisms for mimicking cell functionalities [112]. The detection of the chikungunya virus has also gained considerable interest, with applications relying on gold and iron oxide nanocomposite systems [113] and retroviral-based virus-like particles in magnetic-bead-based platforms [114]. Other studies have focused on the detection of adenoviruses and rotaviruses using magnetic particles functionalized with monoclonal antibodies [115,116].

Moreover, the detection of bacteria has witnessed considerable advancements through the use of magnetic particles. Thus, Gram-negative bacteria, especially *L. monocytogenes*, have been efficiently captured using vancomycin-functionalized polyethylene glycol-modified magnetic nanoparticles, due to their surface moieties [117]. Moreover, a multiplex method for the detection of *Staphylococcus aureus,* methicillin-resistant *Staphylococcus aureus*, and *Klebsiella pneumoniae* was reported using systems based on magnetic particles and fluorescent nanoparticles, namely quantum dots, proving its potential in detecting very low concentrations [118]. Other methods for specifically detecting methicillin-resistant *Staphylococcus aureus* involve the use of dimer-like iron oxide–silver hybrid nanoparticles, which could also be applied for the effective treatment of infectious diseases through their bactericidal properties [119]. Additionally, *Staphylococcus aureus* could be detected using magnetic particles coated with a layer of molecularly imprinted polymer [120], aptamer-coated magnetic beads and vancomycin-stabilized fluorescent gold nanoclusters [121], or aptamer-coated magnetic nanoprobes and vancomycin-functionalized platinum nanoparticles [122]. Other bacteria used as models for the molecular diagnosis of infectious diseases using magnetic particles include *Escherichia coli* and *Salmonella typhimurium* [123,124,125]. Specifically, a new bionanosensor has been developed for the isolation and detection of bacteria through magnetic separation and surface enhanced Raman scattering, using lectin-functionalized magnetic nanoparticles [123]. Furthermore, other systems include 2-nitrodopamine-modified magnetic particles anchored on reduced graphene oxide nanocomposites for both the detection and elimination of *Escherichia coli* in urinary tract infections [124] and polyethylene imine- and amine-functionalized iron oxide particles coated with silica, proving their potential for the effective capture of negative-charge bacterial cells [125].

Antifungal activity against *Candida* spp. has been evaluated using a liquid crystalline system containing iron oxide magnetic nanoparticles for the controlled release of propolis in the intra-periodontal pocket [126]. Additionally, a complex system consisting of antigen-immobilized magnetic particles, disposable electrochemical cells, hardware, and software has been developed as a portable point of care platform for the serologic diagnosis of infectious diseases. This system is of key interest in underdeveloped or isolated areas where there is an acute lack of trained analysts or facilities [127].

## 5. Conclusions and Future Perspectives

Molecular diagnosis has emerged as an innovative field encompassing a variety of methods and techniques to identify variations at the gene, DNA, RNA, or protein levels. Although there have been tremendous advancements in this area since the discovery of the Sanger sequencing method in 1977, these methods are still limited by low sensitivity and specificity, laboriousness, and time consumption. The aim of this paper is to emphasize the importance of magnetic particles’ application in molecular diagnosis, for sample preparation and targeted analyte isolation, purification, and extraction. The basic principle for using magnetic particles in sample preparation is based on the attachment of the targeted analytes onto the particles using specific ligands and receptors, and their subsequent magnetic separation by applying an external magnetic field. Such systems are of key interest in the diagnosis of cancer and infectious diseases, as they allow for the detection of extremely low concentrations of biomarkers and pathogens in biological fluids. There is still room for improvement, as there is a fundamental need to discover new, cancer-specific biomarkers that could be detected using magnetic molecular diagnosis. Thus, cancer screening and early stage detection could be improved and could possibly lead to the cure of cancer. Additionally, more pathogen-specific methods for diagnosing infectious diseases could lead to enhanced diagnostic tools that could prevent further spreading. In this manner, point of care devices could be widely implemented as standard tools to assess various disorders of genetic, environmental, or microbial origins.
